# Validation of knee osteoarthritis case identification algorithms in a large electronic health record database

**DOI:** 10.1016/j.ocarto.2021.100229

**Published:** 2021-12-13

**Authors:** Michelle S. Yau, Maureen Dubreuil, Shanshan Li, Vibha Inamdar, Christine Peloquin, David T. Felson

**Affiliations:** a Hinda and Arthur Marcus Institute for Aging Research, Hebrew SeniorLife, Boston, MA, USA; b Department of Medicine, Beth Israel Deaconess Medical Center and Harvard Medical School, Boston, MA, USA; c Section of Rheumatology, Department of Medicine, Boston University School of Medicine, Boston, MA, USA; d University of Manchester, Manchester, UK

**Keywords:** Knee osteoarthritis, Electronic health records, Validation, Algorithms

## Abstract

**Purpose:**

To facilitate studies of knee osteoarthritis (OA) in large databases, case finding algorithms with high levels of diagnostic performance are needed.

**Methods:**

From a UK general practitioner (GP) practice derived database, we selected adults ages 40–90 years meeting algorithms that included various combinations of codes for knee OA or knee pain and imaging. The GP for each patient was mailed a questionnaire to assess the cause of knee pain and provide knee x-ray and/or MRI findings. We considered knee pain with x-ray and/or MRI findings consistent with OA the gold standard. We calculated positive predictive values (PPV) and sensitivity for case identification algorithms.

**Results:**

Of 100 questionnaires sent, 93 were returned; we excluded 8 subjects who had other rheumatic disorders or total knee replacements. Among those with one code for OA, the PPV was 64% (95% CI = 49%–79%) and it increased to 92% (95% CI = 76%–100%) when two or more OA codes over six months were required. The increase in PPV was accompanied by a drop in sensitivity from 44% (95% CI = 31%–57%) to 19% (95% CI = 9%–30%). Use of one pain code yielded similar results to use of one OA code. Requiring two or more knee pain codes over six months yielded a PPV of 68% (95% CI = 49%–88%) and sensitivity of 26% (95% CI = 15%–38%).

**Discussion:**

A case identification algorithm requiring two or more knee OA codes yielded the highest PPV at the cost of reduced sensitivity. Tradeoffs between PPV and sensitivity will need to be weighed alongside study goals when selecting a case identification algorithm.

## 1. Introduction

Osteoarthritis (OA) is the most common form of arthritis worldwide. For knee OA, one of the most common and symptomatic sites for disease, there is no medical treatment that delays disease progression and patients often undergo knee replacement surgery. Rates of total joint replacement surgeries are increasing exponentially in the United States with estimates that over 1.6 million knee replacements will be performed annually by the year 2030 [1]. The increasing rates of joint replacement surgeries for OA and the costs of disability from OA are growing causes of concern.

Large observational studies, including the Multicenter Osteoarthritis Study (MOST) and Osteoarthritis Initiative (OAI) have provided many new insights into OA, but are limited to a well-defined cohort, often selected for OA risk factors. To further accelerate the discovery of novel OA risk factors and insights into management strategies, even larger studies will be needed. Many newly developed large-scale resources like the United Kingdom (UK) Biobank and All of Us are population-based studies that are linked to electronic health records (EHRs), potentially providing information on thousands of persons with OA. Additionally, EHR data may complement other data sources by providing an assessment of real-world medication utilization and effectiveness. These resources provide much larger sample sizes than can be reasonably recruited in an observational study or clinical trial.

To the best of our knowledge, there has been only one published study examining an algorithm to identify knee OA in claims or EHR databases. The one study with validation of knee OA against physician diagnosis ICD10 code was unusual in that it studied older men and women who, as part of a cohort study, had been asked about frequent knee pain and obtained knee radiographs, gold standards for OA diagnosis [2]. The positive predictive value (PPV) for a single physician diagnosis of OA in this cohort was 88%. There has also been one study of OA that combined knee, hip, and hand OA [3] and another focusing on hip OA [4]. In the study combining hand, knee and hip OA, positive predictive values (PPVs) were >80% for a single report of OA but even higher PPVs if two or more codes for OA are used [3]. The high PPVs from these studies may have been because for joints studied, most did not require imaging evidence of OA to diagnose disease. Another reason for the high PPV is that only OA codes were tested even though some patients with OA are treated without such codes ever used.

Previous studies of knee OA in EHR and claims databases have often only included one diagnostic code to identify persons with OA [5–7]. Whether this is sufficient to accurately identify OA is unclear. Single ICD-9 or ICD-10 codes for other forms of arthritis have had variable predictive value. For example, in both rheumatoid arthritis (RA) and ankylosing spondylitis, a single ICD-9 code has a low PPV [8,9]. Validation studies have suggested that for RA, cases need at least one diagnostic code plus documentation of RA treatment, or at least two diagnostic codes separated by a period of time.

Knee OA cases may be more challenging to identify in large databases than rheumatic diseases for which widely used effective drug therapies can be used to identify cases. Further, chronic knee pain in middle aged and older persons is treated the same way as knee OA and imaging may not be obtained. It is unclear whether those with chronic knee pain should be labeled as having knee OA. Most but not all criteria for knee OA require the presence of knee pain and some structural evidence of OA, usually evidenced by imaging.

We sought to evaluate case identification algorithms to identify persons with knee OA within The Health Improvement Network (THIN), a large EHR database from the UK.

### 1.1. Methods

The Health Improvement Network (THIN) (now called IQVIA Medical Research Data (IMRD)) is an anonymized EHR database collected from general practitioners (GPs) in the United Kingdom (UK) including data on over 11 million patients, with over 45 million patient-years of data (http://www.epic-uk.org/). THIN data represent routine medical practice in a population-based setting. The dataset includes demographics, details from GP visits, specialists’ reports and hospital admissions, test results, height, weight, blood pressure, and smoking status.

We randomly selected 100 patients from THIN who were ages 40–90 years, enrolled with a GP for at least 1 year between January 1, 2000 to May 31, 2015, and had their first diagnostic code for knee OA or knee pain within the assessment period (Supplementary Table 1). To test common strategies for case identification, subjects also had to meet one of three criteria: (1) one or more diagnostic code(s) for knee OA, (2) one or more diagnostic codes for knee OA and a knee x-ray or magnetic resonance images (MRI) within 2 years of knee OA diagnosis, (3) one or more diagnostic codes for knee pain with a knee x-ray or MRI within 2 years of knee pain diagnosis. If the patient had 2 or more codes for OA and/or knee pain, these needed to be separated by at least 7 days. The final selection included 25 subjects who had OA with imaging, 25 subjects who had OA without separate codes for imaging, and 50 subjects who had knee pain with imaging. Subjects were excluded from analyses if they had diagnostic codes for rheumatoid arthritis, pseudogout, gout, or psoriatic arthritis requiring two diagnostic codes separated by at least one week or a code for total knee replacement before the code for OA or knee pain (Supplementary Table 1). Questionnaires were sent to the subjects’ GPs (Supplementary Table 2) to assess the cause of knee pain and whether it was present for more than 6 weeks and to obtain knee x-ray and/or MRI findings. This study received Institutional Review Board approval from Boston University and was determined not human subjects research (H-35312).

#### 1.1.1. Development of OA case identification algorithms

Based on strategies used for other types of arthritis, we developed several case identification algorithms for knee OA. In a previous validation study of ankylosing spondylitis, two diagnostic codes yielded the highest PPV of the tested strategies [8]. We therefore considered algorithms that tested knee OA and knee pain diagnostic codes separated by 7 days over a period of 6 months and 12 months. We created eight algorithms to be tested against the gold standard: 1) one OA code, 2) one pain code, 3) two or more OA codes within six months, 4) two or more pain codes within six months, 5) one or more OA code and one or more pain code within six months, 6) two or more OA codes within 12 months, 7) two or more pain codes within 12 months, and 8) one or more OA code and one or more pain code within 12 months. In secondary analyses, we assessed the performance of these algorithms with the additional requirement of having a documented x-ray and/or MRI imaging within two years of the knee OA or knee pain diagnoses.

We defined the gold standard measure of knee OA as the presence of knee pain and a knee x-ray or MRI demonstrating OA. This conforms with the American College of Rheumatology (ACR) classification criteria for knee OA [10]. Knee pain was defined as a read code for knee pain (1M10.00), anterior knee pain (1M12.00 or N094W00), knee joint pain (N094611), or arthralgia of knee (N094M00). Knee X-ray and MRI reports were obtained from either text comments associated with the procedure code or copies of imaging reports provided by GPs. Presence of knee OA was determined by review of knee x-ray and MRI reports. These reports were reviewed by an experienced rheumatology fellow and senior rheumatologist (VI and DF). Images were not available for review. Presence of knee OA was defined as having definite osteophytes or joint space narrowing or report of presence of OA or degenerative arthritis according to the radiologist’s impression on the imaging report. Findings of sclerosis or tibial spiking alone were not sufficient to meet the criteria for knee OA. Imaging report reviewers were trained on a test set of 10 randomly selected reports until agreement was 90% or higher. Inter-reader reliability was 100% and intra-reader reliability was 100%. Interpretation of imaging reports was performed blinded to each subject’s case status, demographics and other clinical factors. Patients who did not have imaging codes often had reports from the GPs that described imaging findings.

#### 1.1.2. Statistical analyses

We assessed each algorithm defined above relative to the gold standard measure for the presence or absence of person-level knee OA. We calculated the positive predictive value (PPV) and sensitivity and corresponding 95% confidence intervals for each algorithm using the ‘senspec’ option in PROC FREQ (PPV = % true positives/test positives; sensitivity is: of those with disease, % who test positive). All analyses were conducted using SAS 9.4 (Cary, NC).

### 1.2. Results

Among the 100 subjects whose GPs received questionnaires, 93 questionnaires were completed and returned. Of these, four subjects were excluded for having gout, two subjects were excluded for having rheumatoid arthritis, and one subject was excluded for having a total knee replacement prior to the first OA or pain code. Additionally, one subject was excluded due to not having enough data needed to classify the subject according to the gold standard, leaving 85 subjects for analyses. Mean (Standard deviation (SD)) age was 63.8 (11.1) and about 53% were women (Table 1). There were 60% with one or more OA codes, 78% with one or more pain codes, and 73% with one or more imaging codes. The frequency of knee OA as defined by the gold standard was 67%.

Among subjects who had only one OA or pain code, the PPV was 64.1% (95% CI = 49.0%–79.2%) and 65.9% (95% CI = 51.9%–79.9%), respectively (Table 2). Sensitivities for one OA or pain code were 43.9% (95% CI = 31.0%–56.7%) and 50.9% (95% CI = 37.9%–63.9%), respectively. When we required two or more OA codes within 6 months, the PPV was 91.7% (95% CI = 76.0%–100.0%) and the sensitivity was 19.3 (95% CI = 9.1%–29.5%). When we required two or more pain codes within 6 months, the PPV was 68.2% (95% CI = 48.7%–87.6%) and the sensitivity was 26.3% (95% CI = 14.9%–37.7%). When we required one or more OA codes and one or more pain codes within 6 months, the PPV was 71.9% (95% CI = 56.3%–87.5%) and the sensitivity was 40.4% (95% CI = 27.6%–53.1%). Extending the time-period between diagnostic codes to 12 months did not substantially change results.

We repeated analyses among individuals who had documented imaging within 2 years of a knee OA or knee pain diagnostic code. Of the 85 subjects included in the analyses, 62 subjects had separate codes that identified knee x-ray or MRI imaging. Among these individuals who had only one OA or pain code, the PPV was 73.7% (95% CI = 53.9%–93.5%) and 67.6% (95% CI = 52.5%–82.7%), respectively (Table 3). Sensitivities for one OA or pain code were 32.6% (95% CI = 18.6%–46.6%) and 58.1% (95% CI = 43.4%–72.9%), respectively. When we required two or more codes within 6 months or 12 months among individuals with knee imaging, the estimates for PPV and sensitivity remained largely similar to estimates obtained among all subjects regardless of whether they had imaging or not.

Our patient selection included 25 individuals who had no codes for imaging. For more than half of these individuals, imaging results were either present in GP notes or reported in the GP questionnaires that were sent. While numbers were small, the PPVs for OA and pain diagnostic codes among these patients were lower for those with one code (mean 56%) compared to those with two or more codes (71%), similar to findings among those who had codes for imaging.

### 1.3. Discussion

In a validation study testing diagnostic algorithms for knee OA, we found that, like other rheumatic diseases, an algorithm that required two diagnostic codes at least 7 days apart for knee OA had a higher PPV than algorithms requiring only a single diagnostic code. If we added those with only knee pain to those with knee OA, we captured more persons with OA, increasing sensitivity, but the PPV did not change.

While diagnostic test performance may differ by setting and country (see below), our study raises questions about the validity of studies that have used EHR and claims databases to evaluate risk factors for OA or OA outcomes, especially those relying on one OA code for diagnosis [5–7]. Low PPVs for one OA code or one pain code indicate that many identified OA cases do not actually have OA or at least have not had imaging studies that would document their OA. The distinction is important because many EHR-based studies will be used for health services research, epidemiologic or genetic studies that seek to identify relevant biology that can be used to identify new treatment targets. Our results are similar to those for other rheumatic disorders including RA and spondyloarthritis, which have reported that two diagnostic codes for the disease have a higher predictive value than one code. PPVs over 70% seen in this study are higher than has been reported in studies of other rheumatic diseases using algorithms that are widely used. It is likely that PPV’s for OA and other musculoskeletal disorders vary from country to country and setting to setting depending on how and where diagnosis coding is carried out. In Sweden where the caring physician enters the code and this is often a physician specializing in a specific set of diseases, PPV’s may be high whereas in a system where an administrative clerk enters the code, PPV’s may be lower; they may be lower in THIN where only general doctors make diagnoses and hospital and specialist diagnoses are not necessarily included.

There have been three other OA studies validating algorithms for identifying OA cases. One focused on knee OA and, as noted earlier, had an unusual design with a specific cohort that already had answered knee pain questions and obtained knee x-rays. In a study of hip OA based in the UK Clinical Practice Research Datalink, which reported a PPV of 79% [4], at least one diagnosis of hip OA was required although there was no comparison of single vs. multiple codes and diagnoses were confirmed with hip pain or stiffness as an alternative to x-ray confirmation. In the other study of hand, knee and hip OA in which imaging was not required for case definition for hip and hand OA [3], PPVs ranged from 82 to 100%, the latter present when more than one diagnostic code was used. The authors noted a drop in sensitivity with the requirement for more than one code but recommended it nonetheless, given its higher PPV. Given the insensitivity of x-ray changes in early OA, these studies reported higher PPVs than our study likely because the gold standard used was not based on x-ray and/or MRI confirmed OA. Pain is a common symptom for OA and other musculoskeletal conditions, making it much easier to achieve high PPVs when imaging is not required as part of the gold standard and may result in less accurate results.

Among important limitations was the small size of our sample. This was driven by the cost of acquiring additional data on persons from the THIN data which limited our numbers. High PPVs may be driven by the high prevalence of OA in our sample and may be different in other samples with a lower prevalence of OA. Despite these limitations, we provide new data from THIN that provides a gold standard assessment of OA needed to assess the use of EHR codes for identifying OA. There are few EHR data sources that provide a gold standard assessment of OA, which is time intensive, requiring clinical chart review or ascertainment of additional clinical data as was done in this study. Larger studies will be needed to confirm our results, but we expect such findings will be similar given the consistency of our results with other rheumatic disorders.

Another possible limitation is that we required knee pain and presence of knee x-ray and/or MRI findings consistent with OA to meet the gold standard definition. Importantly, OA may be diagnosed and treated without imaging. In fact, imaging is not recommended in current guidelines mostly because treatment of chronic knee pain and treatment of OA are similar. Further, many persons with chronic knee pain who failed to meet our criteria, which required imaging confirmation of knee OA, may have OA [11]. This raises challenging questions about whether to require imaging evidence of OA in large-scale studies. In secondary analyses, we showed that the addition of imaging findings did not substantially change PPVs compared to the same case identification algorithms used without the requirement of imaging; some of these individuals had imaging but it was not required in the case finding algorithm. Knee pain symptoms can be transient or mild and may reflect other disorders and not OA, which is why we required the presence of imaging evidence in our gold standard definition. The decision to require imaging evidence of OA in a claims or EHR based OA study depends on the question posed and whether it requires that cases have unequivocal disease. If so, imaging evidence of disease would be preferred.

Our study provides new information that should inform studies of claims and EHR-based studies of OA. Using one claim or diagnosis code for OA may leave many persons misclassified and may compromise the validity of analyses related to OA. However, if potential misclassification is not a concern and a study aims to identify as many OA cases as possible, a single knee pain or OA code may be used since it has higher sensitivity. Whether to prioritize PPV or sensitivity depends on the study question. A two-phase approach may be used, where the first phase prioritizes sample size over misclassification, yielding findings that are brought forward to a second phase that prioritizes PPV over sample size. This may help yield meaningful results.

In conclusion, large data base analyses targeting OA should base their case algorithm on PPV and the sensitivity of the case finding strategy. In settings where the PPV for one diagnostic code is low, use of two diagnoses of OA separated by at least 7 days may be needed to ensure a high PPV. Adding knee pain as diagnostic code has tradeoffs, with improved sensitivity and number of cases but this may lead to a drop in PPV. More studies on the optimal strategy to identify knee OA cases in large datasets are needed including whether and how these are affected by the way the data are coded and whether imaging is critical to the case definition.

## Supplementary Material

Supplementary Material

## Figures and Tables

**Table 1:** Sample characteristics for individuals with the gold standard assessment (n = 85).

Mean age in years (SD)	63.8 (11.1)
% Women	53
% with one or more OA codes	60
% with one or more pain codes	78
% with one or more imaging codes	73
% OA according to gold standard	67

**Table 2:** Estimates of positive predictive value and sensitivity among individuals with the gold standard assessment, regardless of imaging status (n = 85).

Definition	Number satisfying definition	Number with OA by gold standard	PPV (95% CI)	Sensitivity (95% CI)
One code^a^				
OA code	39	25	64.1 (49.0, 79.2)	43.9 (31.0, 56.7)
knee pain code^b^	44	29	65.9 (51.9, 79.9)	50.9 (37.9, 63.9)
≥2 codes within 6 months				
≥2 OA codes	12	11	91.7 (76.0,100.0)	19.3 (9.1, 29.5)
≥2 knee pain codes^b^	22	15	68.2 (48.7, 87.6)	26.3 (14.9, 37.7)
≥1 OA and ≥1 knee pain code	32	23	71.9 (56.3, 87.5)	40.4 (27.6, 53.1)
≥2 codes within 12 months				
≥2 OA codes	14	12	85.7 (67.4, 100.0)	21.1 (10.5, 31.6)
≥2 knee pain codes^b^	30	20	66.7 (49.8, 83.5)	35.1 (22.7, 47.5)
≥1 OA and ≥1 knee pain code	32	23	71.9 (56.3, 87.5)	40.4 (27.6, 53.1)

a Exactly one code.

b All those with knee pain codes only had to have imaging ordered.

**Table 3:** Estimates of positive predictive value and sensitivity among individuals with the gold standard assessment and who also were selected because they had imaging (n = 62)^b^.

Definition	Number satisfying definition	Number with OA by gold standard	PPV (95% CI)	Sensitivity (95% CI)
One code^a^				
OA code	19	14	73.7 (53.9, 93.5)	32.6 (18.6, 46.6)
knee Pain code	37	25	67.6 (52.5, 82.7)	58.1 (43.4, 72.9)
≥2 codes within 6 months				
≥2 OA codes	9	8	88.9 (68.4, 100.0)	18.6 (7.0, 30.2)
≥2 knee pain codes	21	14	66.7 (46.5, 86.8)	32.6 (18.6, 46.6)
≥1 OA and ≥1 knee pain code	24	18	75.0 (57.7, 92.3)	41.9 (27.1, 56.6)
≥2 codes within 12 months				
≥2 OA codes	11	9	81.8 (59.0, 100.0)	20.9 (8.8, 33.1)
≥2 knee pain codes	28	19	67.9 (50.6, 85.2)	44.2 (29.3, 59.0)
≥1 OA and ≥1 knee pain code	24	18	75.0 (57.7, 92.3)	41.9 (27.1, 56.6)

a Exactly one code.

b As noted in text, many selected without imaging turned out to have imaging with results described in chart review or x-ray reports.
