# Supplementary material for: Validation of knee osteoarthritis case identification algorithms in a large electronic health record database
Source: Osteoarthr Cartil Open. 2021 Dec 13;4(1):100229. doi: 10.1016/j.ocarto.2021.100229 (PMC9122022; doi:10.1016/j.ocarto.2021.100229)
Supplement: Multimedia component 1 [file mmc1.docx]

**Supplementary Material**

**Supplementary Table 1.** Codes used to define knee OA, knee pain, knee imaging, gout, pseudogout, rheumatoid arthritis, and total knee replacement

| Knee OA |
| --- |
| N051B00 Primary gonarthrosis, bilateral |
| N052A00 Post-traumatic gonarthrosis, bilateral |
| N052C00 Post-traumatic gonarthrosis, unilateral |
| N05z611 Knee osteoarthritis NOS |
| N05zL00 Osteoarthritis NOS, of knee |
| Nyu2500 [X]Other primary gonarthrosis |
| Nyu2511 [X] Unilateral primary gonarthrosis |
| Nyu2700 [X]Other secondary gonarthrosis, bilateral |
| Nyu2800 [X]Other secondary gonarthrosis |
| Nyu2811 [X] Unilateral secondary gonarthrosis |
|  |
| Knee Pain |
| 1M10.00 Knee pain |
| 1M12.00 Anterior knee pain |
| N094611 Knee joint pain |
| N094M00 Arthralgia of knee |
| N094W00 Anterior knee pain |
|  |
| Knee Image |
| 52A..13 Knee X-ray |
| 52A7.00 Plain X-ray knee |
| 52A7000 Plain X-ray knee normal |
| 52A7100 Plain X-ray knee abnormal |
| 52AB.00 Stress X-ray knee |
| 569L.00 Magnetic resonance imaging of knee |
| 569L.11 MRI of knee |
|  |
| Gout |
| 1443.00 H/O: gout |
| 669..00 Gout monitoring |
| 6692.00 Follow-up gout assessment |
| 6693.00 Joints gout affected |
| 6697.00 Gout associated problems |
| 6698.00 Gout drug side effects |
| 6699.00 Gout treatment changed |
| 669Z.00 Gout monitoring NOS |
| C34..00 Gout |
| C340.00 Gouty arthropathy |
| C342.00 Idiopathic gout |
| C343.00 Lead-induced gout |
| C344.00 Drug-induced gout |
| C345.00 Gout due to impairment of renal function |
| C346.00 Acute exacerbation of gout |
| C34y.00 Other specified gouty manifestation |
| C34y000 Gouty tophi of ear |
| C34y200 Gouty tophi of other sites |
| C34y500 Gouty tophi of hand |
| C34yz00 Other specified gouty manifestation NOS |
| C34z.00 Gout NOS |
| N023.00 Gouty arthritis |
| N023000 Gouty arthritis of unspecified site |
| N023100 Gouty arthritis of the shoulder region |
| N023200 Gouty arthritis of the upper arm |
| N023300 Gouty arthritis of the forearm |
| N023400 Gouty arthritis of the hand |
| N023500 Gouty arthritis of the pelvic region and thigh |
| N023600 Gouty arthritis of the lower leg |
| N023700 Gouty arthritis of the ankle and foot |
| N023800 Gouty arthritis of toe |
| N023x00 Gouty arthritis of multiple sites |
| N023y00 Gouty arthritis of other specified site |
| N023z00 Gouty arthritis NOS |
| Nyu1700 [X]Other secondary gout |
|  |
| Pseudogout |
| N02..14 Pseudogout |
|  |
| Psoriatic arthritis |
| M160.00 Psoriatic arthropathy |
| M160.11 Psoriatic arthritis |
| M160000 Psoriasis spondylitica |
| M160100 Distal interphalangeal psoriatic arthropathy |
| M160200 Arthritis mutilans |
| M160z00 Psoriatic arthropathy NOS |
| Nyu1300 [X]Other psoriatic arthropathies |
|  |
| Rheumatoid arthritis |
| 38DZ.00 Disease activity score in rheumatoid arthritis |
| 38DZ000 Disease activity score 28 joint in rheumatoid arthritis |
| G5y8.00 Rheumatoid myocarditis |
| G5yA.00 Rheumatoid carditis |
| H570.00 Rheumatoid lung |
| N04..00 Rheumatoid arthritis and other inflammatory polyarthropathy |
| N040.00 Rheumatoid arthritis |
| N040000 Rheumatoid arthritis of cervical spine |
| N040100 Other rheumatoid arthritis of spine |
| N040200 Rheumatoid arthritis of shoulder |
| N040300 Rheumatoid arthritis of sternoclavicular joint |
| N040400 Rheumatoid arthritis of acromioclavicular joint |
| N040500 Rheumatoid arthritis of elbow |
| N040600 Rheumatoid arthritis of distal radio-ulnar joint |
| N040700 Rheumatoid arthritis of wrist |
| N040800 Rheumatoid arthritis of MCP joint |
| N040900 Rheumatoid arthritis of PIP joint of finger |
| N040A00 Rheumatoid arthritis of DIP joint of finger |
| N040B00 Rheumatoid arthritis of hip |
| N040C00 Rheumatoid arthritis of sacro-iliac joint |
| N040D00 Rheumatoid arthritis of knee |
| N040E00 Rheumatoid arthritis of tibio-fibular joint |
| N040F00 Rheumatoid arthritis of ankle |
| N040G00 Rheumatoid arthritis of subtalar joint |
| N040H00 Rheumatoid arthritis of talonavicular joint |
| N040J00 Rheumatoid arthritis of other tarsal joint |
| N040K00 Rheumatoid arthritis of 1st MTP joint |
| N040L00 Rheumatoid arthritis of lesser MTP joint |
| N040M00 Rheumatoid arthritis of IP joint of toe |
| N040N00 Rheumatoid vasculitis |
| N040P00 Seronegative rheumatoid arthritis |
| N040Q00 Rheumatoid bursitis |
| N040S00 Rheumatoid arthritis - multiple joint |
| N040T00 Flare of rheumatoid arthritis |
| N041.00 Felty's syndrome |
| N042.00 Other rheumatoid arthropathy + visceral/systemic involvement |
| N042100 Rheumatoid lung disease |
| N042z00 Rheumatoid arthropathy + visceral/systemic involvement NOS |
| N047.00 Seropositive errosive rheumatoid arthritis |
| N04X.00 Seropositive rheumatoid arthritis, unspecified |
| N04y000 Rheumatoid lung |
| N04y011 Caplan's syndrome |
| N04y012 Fibrosing alveolitis associated with rheumatoid arthritis |
|  |
| Total knee replacement |
| 14N3200 H/O knee replacement |
| 7K30.00 Total prosthetic replacement of knee joint using cement |
| 7K30.11 Anametric total replacement of knee joint using cement |
| 7K30.12 Arthroplasty of knee joint using cement |
| 7K30.13 Attenborough total replacement of knee joint using cement |
| 7K30.14 Autophor arthroplasty of knee joint using cement |
| 7K30.15 Cavendish total replacement of knee joint using cement |
| 7K30.16 Charnley total replacement of knee joint using cement |
| 7K30.17 Deane total replacement of knee joint using cement |
| 7K30.18 Denham total replacement of knee joint using cement |
| 7K30.19 Freeman total replacement of knee joint using cement |
| 7K30.1A Geomedic total replacement of knee joint using cement |
| 7K30.1B Geometric total replacement of knee joint using cement |
| 7K30.1C Guepar hinge replacement of knee joint using cement |
| 7K30.1D Gunston total replacement of knee joint using cement |
| 7K30.1E Herbert total replacement of knee joint using cement |
| 7K30.1F Ilch total replacement of knee joint using cement |
| 7K30.1G Irving total replacement of knee joint using cement |
| 7K30.1H Liverpool total replacement of knee joint using cement |
| 7K30.1I Manchester total replacement of knee joint using cement |
| 7K30.1J Marmor total replacement of knee joint using cement |
| 7K30.1K McKee arthroplasty of knee joint using cement |
| 7K30.1L Melbourne total replacement of knee joint using cement |
| 7K30.1M Platt arthroplasty of knee joint using cement |
| 7K30.1N Polycentric total replacement of knee joint using cement |
| 7K30.1O Pretoria arthroplasty of knee joint using cement |
| 7K30.1P Sheehan total replacement of knee joint using cement |
| 7K30.1Q Shiers total replacement of knee joint using cement |
| 7K30.1R Stanmore total replacement of knee joint using cement |
| 7K30.1S Swanson total replacement of knee joint using cement |
| 7K30.1T Uci total replacement of knee joint using cement |
| 7K30.1U Wallidus hinge arthroplasty of knee joint using cement |
| 7K30.1V TKR -Total prosthetic replacement of knee joint using cement |
| 7K30000 Primary cemented total knee replacement |
| 7K30y00 Total prosthetic replacement of knee joint using cement OS |
| 7K30z00 Total prosthetic replacement of knee joint using cement NOS |
| 7K31.00 Total prosthetic replacement of knee joint not using cement |
| 7K31.11 Arthroplasty of knee joint not using cement |
| 7K31.12 TKR - Total prosthetic replacement knee joint without cement |
| 7K31000 Primary uncemented total knee replacement |
| 7K31y00 Total prosthetic replacement knee joint not using cement OS |
| 7K31z00 Total prosthetic replacement knee joint not using cement NOS |
| 7K32.00 Other total prosthetic replacement of knee joint |
| 7K32.11 Other arthroplasty of knee joint |
| 7K32.12 TKR - Other total prosthetic replacement of knee joint |
| 7K32000 Primary total knee replacement NEC |
| 7K32011 Primary hybrid total knee replacement NEC |
| 7K32600 Prosthetic arthroplasty of the patellofemoral joint |
| 7K32y00 Other total prosthetic replacement of knee joint OS |

**Supplementary Table 2**. Questionnaire items sent to subjects’ general practitioners

| **Description** |
| --- |
| Questionnaire reference number |
| Patient identification number |
| Physician practice ID number |
| Year of birth |
| Sex |
| Q1: Painful knee OA? 0= no, 1=yes |
| Q1: Text comments |
| Q2: Date of knee pain onset. Enter the Month/4 digit year as written. If no month, enter 00/year. If blank, enter . (period) |
| Q3: Date of first knee pain treatment. Enter the Month/4 digit year as written. If no month, enter 00/year. If blank, enter . (period) |
| Q3: Text comments |
| Q4: Knee pain present at first visit to practice? Enter 0=no, 1=yes. If not known, enter . (period) |
| Q5: Knee pain present for more than 6 weeks? Enter 0=no, 1=yes. If not known, enter . (period) |
| Q6: 1=xray, 2=mri, 3=neither, 4=can't provide |
| Q6: text summary of report |
| Text from x-ray report |
| Text from MRI report |
| Q7: Did later xray show OA? 1=N/A, 2=yes, 3=no, 4=unknown |
| Date for report in Q7 |
| Text of any later reports |
| General data notes |
