# Supplementary material for: Validation of knee osteoarthritis case identification algorithms in a large electronic health record database
Source: Osteoarthr Cartil Open. Author manuscript; Available in PMC 2022 May 20. (PMC9122022; doi:10.1016/j.ocarto.2021.100229)
Supplement: Supplementary file 1 [file NIHMS1807226-supplement-Supplementary_Material.docx]

**Supplementary Material**

**Supplementary Table 1.** Codes used to define knee OA, knee pain, knee imaging, gout, pseudogout, rheumatoid arthritis, and total knee replacement

| Knee OA |
| --- |
| N051B00     Primary gonarthrosis, bilateral |
| N052A00     Post-traumatic gonarthrosis, bilateral |
| N052C00     Post-traumatic gonarthrosis, unilateral |
| N05z611     Knee osteoarthritis NOS |
| N05zL00     Osteoarthritis NOS, of knee |
| Nyu2500     [X]Other primary gonarthrosis |
| Nyu2511     [X] Unilateral primary gonarthrosis |
| Nyu2700     [X]Other secondary gonarthrosis, bilateral |
| Nyu2800     [X]Other secondary gonarthrosis |
| Nyu2811     [X] Unilateral secondary gonarthrosis |
|  |
| Knee Pain |
| 1M10.00     Knee pain |
| 1M12.00     Anterior knee pain |
| N094611     Knee joint pain |
| N094M00     Arthralgia of knee |
| N094W00     Anterior knee pain |
|  |
| Knee Image |
| 52A..13     Knee X-ray |
| 52A7.00     Plain X-ray knee |
| 52A7000     Plain X-ray knee normal |
| 52A7100     Plain X-ray knee abnormal |
| 52AB.00     Stress X-ray knee |
| 569L.00     Magnetic resonance imaging of knee |
| 569L.11     MRI of knee |
|  |
| Gout |
| 1443.00     H/O: gout |
| 669..00     Gout monitoring |
| 6692.00     Follow-up gout assessment |
| 6693.00     Joints gout affected |
| 6697.00     Gout associated problems |
| 6698.00     Gout drug side effects |
| 6699.00     Gout treatment changed |
| 669Z.00     Gout monitoring NOS |
| C34..00     Gout |
| C340.00     Gouty arthropathy |
| C342.00     Idiopathic gout |
| C343.00     Lead-induced gout |
| C344.00     Drug-induced gout |
| C345.00     Gout due to impairment of renal function |
| C346.00     Acute exacerbation of gout |
| C34y.00     Other specified gouty manifestation |
| C34y000     Gouty tophi of ear |
| C34y200     Gouty tophi of other sites |
| C34y500     Gouty tophi of hand |
| C34yz00     Other specified gouty manifestation NOS |
| C34z.00     Gout NOS |
| N023.00     Gouty arthritis |
| N023000     Gouty arthritis of unspecified site |
| N023100     Gouty arthritis of the shoulder region |
| N023200     Gouty arthritis of the upper arm |
| N023300     Gouty arthritis of the forearm |
| N023400     Gouty arthritis of the hand |
| N023500     Gouty arthritis of the pelvic region and thigh |
| N023600     Gouty arthritis of the lower leg |
| N023700     Gouty arthritis of the ankle and foot |
| N023800     Gouty arthritis of toe |
| N023x00     Gouty arthritis of multiple sites |
| N023y00     Gouty arthritis of other specified site |
| N023z00     Gouty arthritis NOS |
| Nyu1700     [X]Other secondary gout |
|  |
| Pseudogout |
| N02..14     Pseudogout |
|  |
| Psoriatic arthritis |
| M160.00     Psoriatic arthropathy |
| M160.11     Psoriatic arthritis |
| M160000     Psoriasis spondylitica |
| M160100     Distal interphalangeal psoriatic arthropathy |
| M160200     Arthritis mutilans |
| M160z00     Psoriatic arthropathy NOS |
| Nyu1300     [X]Other psoriatic arthropathies |
|  |
| Rheumatoid arthritis |
| 38DZ.00     Disease activity score in rheumatoid arthritis |
| 38DZ000     Disease activity score 28 joint in rheumatoid arthritis |
| G5y8.00     Rheumatoid myocarditis |
| G5yA.00     Rheumatoid carditis |
| H570.00     Rheumatoid lung |
| N04..00     Rheumatoid arthritis and other inflammatory polyarthropathy |
| N040.00     Rheumatoid arthritis |
| N040000     Rheumatoid arthritis of cervical spine |
| N040100     Other rheumatoid arthritis of spine |
| N040200     Rheumatoid arthritis of shoulder |
| N040300     Rheumatoid arthritis of sternoclavicular joint |
| N040400     Rheumatoid arthritis of acromioclavicular joint |
| N040500     Rheumatoid arthritis of elbow |
| N040600     Rheumatoid arthritis of distal radio-ulnar joint |
| N040700     Rheumatoid arthritis of wrist |
| N040800     Rheumatoid arthritis of MCP joint |
| N040900     Rheumatoid arthritis of PIP joint of finger |
| N040A00     Rheumatoid arthritis of DIP joint of finger |
| N040B00     Rheumatoid arthritis of hip |
| N040C00     Rheumatoid arthritis of sacro-iliac joint |
| N040D00     Rheumatoid arthritis of knee |
| N040E00     Rheumatoid arthritis of tibio-fibular joint |
| N040F00     Rheumatoid arthritis of ankle |
| N040G00     Rheumatoid arthritis of subtalar joint |
| N040H00     Rheumatoid arthritis of talonavicular joint |
| N040J00     Rheumatoid arthritis of other tarsal joint |
| N040K00     Rheumatoid arthritis of 1st MTP joint |
| N040L00     Rheumatoid arthritis of lesser MTP joint |
| N040M00     Rheumatoid arthritis of IP joint of toe |
| N040N00     Rheumatoid vasculitis |
| N040P00     Seronegative rheumatoid arthritis |
| N040Q00     Rheumatoid bursitis |
| N040S00     Rheumatoid arthritis - multiple joint |
| N040T00     Flare of rheumatoid arthritis |
| N041.00     Felty's syndrome |
| N042.00     Other rheumatoid arthropathy + visceral/systemic involvement |
| N042100     Rheumatoid lung disease |
| N042z00     Rheumatoid arthropathy + visceral/systemic involvement NOS |
| N047.00     Seropositive errosive rheumatoid arthritis |
| N04X.00     Seropositive rheumatoid arthritis, unspecified |
| N04y000     Rheumatoid lung |
| N04y011     Caplan's syndrome |
| N04y012     Fibrosing alveolitis associated with rheumatoid arthritis |
|  |
| Total knee replacement |
| 14N3200     H/O knee replacement |
| 7K30.00     Total prosthetic replacement of knee joint using cement |
| 7K30.11     Anametric total replacement of knee joint using cement |
| 7K30.12     Arthroplasty of knee joint using cement |
| 7K30.13     Attenborough total replacement of knee joint using cement |
| 7K30.14     Autophor arthroplasty of knee joint using cement |
| 7K30.15     Cavendish total replacement of knee joint using cement |
| 7K30.16     Charnley total replacement of knee joint using cement |
| 7K30.17     Deane total replacement of knee joint using cement |
| 7K30.18     Denham total replacement of knee joint using cement |
| 7K30.19     Freeman total replacement of knee joint using cement |
| 7K30.1A     Geomedic total replacement of knee joint using cement |
| 7K30.1B     Geometric total replacement of knee joint using cement |
| 7K30.1C     Guepar hinge replacement of knee joint using cement |
| 7K30.1D     Gunston total replacement of knee joint using cement |
| 7K30.1E     Herbert total replacement of knee joint using cement |
| 7K30.1F     Ilch total replacement of knee joint using cement |
| 7K30.1G     Irving total replacement of knee joint using cement |
| 7K30.1H     Liverpool total replacement of knee joint using cement |
| 7K30.1I     Manchester total replacement of knee joint using cement |
| 7K30.1J     Marmor total replacement of knee joint using cement |
| 7K30.1K     McKee arthroplasty of knee joint using cement |
| 7K30.1L     Melbourne total replacement of knee joint using cement |
| 7K30.1M     Platt arthroplasty of knee joint using cement |
| 7K30.1N     Polycentric total replacement of knee joint using cement |
| 7K30.1O     Pretoria arthroplasty of knee joint using cement |
| 7K30.1P     Sheehan total replacement of knee joint using cement |
| 7K30.1Q     Shiers total replacement of knee joint using cement |
| 7K30.1R     Stanmore total replacement of knee joint using cement |
| 7K30.1S     Swanson total replacement of knee joint using cement |
| 7K30.1T     Uci total replacement of knee joint using cement |
| 7K30.1U     Wallidus hinge arthroplasty of knee joint using cement |
| 7K30.1V     TKR -Total prosthetic replacement of knee joint using cement |
| 7K30000     Primary cemented total knee replacement |
| 7K30y00     Total prosthetic replacement of knee joint using cement OS |
| 7K30z00     Total prosthetic replacement of knee joint using cement NOS |
| 7K31.00     Total prosthetic replacement of knee joint not using cement |
| 7K31.11     Arthroplasty of knee joint not using cement |
| 7K31.12     TKR - Total prosthetic replacement knee joint without cement |
| 7K31000     Primary uncemented total knee replacement |
| 7K31y00     Total prosthetic replacement knee joint not using cement OS |
| 7K31z00     Total prosthetic replacement knee joint not using cement NOS |
| 7K32.00     Other total prosthetic replacement of knee joint |
| 7K32.11     Other arthroplasty of knee joint |
| 7K32.12     TKR - Other total prosthetic replacement of knee joint |
| 7K32000     Primary total knee replacement NEC |
| 7K32011     Primary hybrid total knee replacement NEC |
| 7K32600     Prosthetic arthroplasty of the patellofemoral joint |
| 7K32y00     Other total prosthetic replacement of knee joint OS |

**Supplementary Table 2**. Questionnaire items sent to subjects’ general practitioners 

| **Description** |
| --- |
| Questionnaire reference number |
| Patient identification number |
| Physician practice ID number |
| Year of birth |
| Sex |
| Q1:  Painful knee OA? 0= no, 1=yes |
| Q1: Text comments |
| Q2: Date of knee pain onset. Enter the Month/4 digit year as written.  If no month, enter 00/year.  If blank, enter . (period) |
| Q3: Date of first knee pain treatment. Enter the Month/4 digit year as written.  If no month, enter 00/year. If blank, enter . (period) |
| Q3: Text comments |
| Q4: Knee pain present at first visit to practice?  Enter 0=no, 1=yes. If not known, enter . (period) |
| Q5: Knee pain present for more than 6 weeks? Enter 0=no, 1=yes. If not known, enter . (period) |
| Q6: 1=xray, 2=mri, 3=neither, 4=can't provide |
| Q6: text summary of report |
| Text from x-ray report |
| Text from MRI report |
| Q7: Did later xray show OA?  1=N/A, 2=yes, 3=no, 4=unknown |
| Date for report in Q7 |
| Text of any later reports |
| General data notes |
